# Dihydroartemisinin attenuates PM-induced lung injury by inhibiting inflammation and regulating autophagy

**DOI:** 10.3389/fpubh.2025.1548224

**Published:** 2025-03-07

**Authors:** Lingjing Liu, Jingli Li, Yincong Xue, Shuying Xie, Nian Dong, Chengshui Chen

**Affiliations:** ^1^Department of Pulmonary and Critical Care Medicine, The First Affiliated Hospital of Wenzhou Medical University, Wenzhou, China; ^2^Department of Pulmonary and Critical Care Medicine, Shaoxing People's Hospital, Shaoxing, Zhejiang, China

**Keywords:** particulate matter (PM), dihydroartemisinin (DHA), lung injury, inflammation, autophagy, nuclear factor-κB (NF-κB)

## Abstract

**Objective:**

The study investigates the effects and mechanisms of dihydroartemisinin (DHA) in mitigating lung injury induced by particulate matter (PM).

**Methods:**

The lung injury model was induced by PM particles *in vivo* and *in vitro*. Hematoxylin and Eosin (H&E) staining was utilized for the detection of the thickening of airway wall and the infiltration of inflammatory cells in mouse lung tissue. The expressions of inflammatory factors were detected in alveolar lavage fluid and cell supernatant. TUNEL (Terminal deoxynucleotidyl transferase dUTP nick end labeling) staining, Caspase-1, Bcl-2-associated X protein (Bax), B-cell lymphoma 2 (Bcl-2), microtubule-associated protein 1 light chain 3-II (LC3-II) and Belcin-1 were used to observe the apoptosis and autophagy related expressions in mouse lung tissue, and p-p65 was detected by immunofluorescence.

**Results:**

H&E staining revealed DHA alleviates PM-induced lung injury *in vivo*. Moreover, DHA reduced IL-6, IL-8, and IL-1β levels by ~50% (*p* < 0.05), highlighting its anti-inflammatory effects. Furthermore, immunohistochemistry showed that DHA treatment inhibited the pro-apoptotic expression of Bax/BCL2 and cleaved-Caspase-3, respectively. In addition, immunofluorescence staining revealed that the LC3-II and Beclin-1 levels dramatically increased in the PM group compared to Control group, but greatly reduced by DHA. Further, we found that DHA inhibited the activation of the NF-KB signaling pathway.

**Conclusion:**

DHA protects against PM-induced lung injury through anti-inflammatory, anti-apoptotic, and autophagy-regulating mechanisms, offering a potential drug option for improving PM-induced lung injury.

## 1 Introduction

Air pollution, driven by the emission of particulate matter (PM) into the atmosphere, presents a formidable threat to global public health (1). Among these particles, fine particulate matter with a diameter < 2.5 micrometers (PM2.5) has garnered particular concern due to its capacity to penetrate deep into the human respiratory system (2). The World Health Organization has underscored that PM2.5 exposure is linked to ~4.2 million annual fatalities (3). Chronic bronchitis, chronic obstructive pulmonary disease (COPD), asthma, and lung cancer are among the prominent respiratory disorders linked to PM2.5 exposure (4). Particles measuring 2.5 micrometers or less successfully bypass nasal filtration, depositing into the deep part of the lung. This accumulation leads to diffuse alveolar damage, resulting in significant disruptions to gas exchange (5). Previous studies have demonstrated that pro-inflammatory cytokines (IL-1, TNF-α, and IL-6), Th0-type cytokine (IL-2), and Th1-type cytokines (IL-12 and IFN-γ) were increased by PM exposed and PM can induced an increase of T cell distribution in lymphocyte and decreased the CD4+/CD8+ ratio, ultimately culminating in pronounced pulmonary inflammatory lesions (6). Moreover, recent researches have highlighted the multifaceted roles of autophagy and apoptosis in modulating both the immune response and pulmonary inflammation during various stages of PM-induced lung injury (7). While autophagy and apoptosis are pivotal processes for maintaining normal lung function, abnormal autophagic activity induced by PM exposure may exacerbate the pathological progression of lung injury by either prompting cell death or initiating apoptosis (8). However, the precise molecular mechanisms underlying the interplay among these processes in PM-induced lung injury remain incompletely understood, leaving a critical gap in developing targeted interventions.

Dihydroartemisinin (DHA), a water-soluble semisynthetic derivative derived from artemisinin, stands out as a pivotal compound in antimalarial therapy (9). Its enhanced oral bioavailability, attributed to the reduction of the carbonyl group at the C-10 position, has significantly augmented its therapeutic potential (10). DHA has a more powerful antimalarial effect than artemisinin, and is widely used as the first-line antimalarial drug. Over the last few years, an increasing number of studies have demonstrated that DHA has antitumor, anti-neurodegeneration, and antifibrotic activities (11–13). Moreover, DHA's proficiency in mitigating oxidative stress and inflammation underscores its crucial role in immune regulation (14). For instance, DHA has been reported to exert anti-inflammatory effects by modulating the Toll-like receptor 4 (TLR4)-mediated NF-κB and MAPK signaling pathways, thereby reducing the production of pro-inflammatory cytokines in macrophages exposed to bacterial endotoxins (9). Li et al. (15) found that DHA effectively alleviates lupus symptoms in BXSB mice, a well-established animal model for systemic lupus erythematosus (SLE), by suppressing the production of tumor necrosis factor α (TNF-α) and impeding the translocation of NF-κB within its signaling pathway. Study has also noted that DHA inhibited the activation of NF-κB pathway by regulating inflammatory mediators such as TNF-α and interleukin 6 (IL-6) to attenuate chronic airway inflammation (16). Besides, it has been reported that DHA notably elevates the levels of superoxide dismutase (SOD) and glutathione (GSH) in rats with bleomycin-induced pulmonary fibrosis, operating through the Nrf2/HO-1 signaling pathway (17).

Despite these findings, the role of DHA in PM-induced lung injury has not been systematically explored, and its potential to modulate key pathological processes such as inflammation, apoptosis, and autophagy in this context remains unclear. This study aims to address these gaps by systematically investigating the protective effects of DHA against PM-induced lung injury in a murine model. We hypothesize whether DHA can improve PM-induced lung injury by reducing the production of inflammatory mediators such as IL-6, IL-8, TNF-α, and inhibiting apoptosis of airway epithelial cells. These findings provide novel insights into the therapeutic mechanisms of DHA, establishing its potential as a multifaceted intervention for PM-related lung injury.

## 2 Materials and methods

### 2.1 Reagents and antibodies

The PM (1649b) was obtained from the Standard Reference Material Program, and this material is certified by the National Institute of Standards and Technology (MD, USA). The PM used in the present study primarily comprised of common components of urban PM, including pesticides, dioxins, polycyclic aromatic hydrocarbons and polychlorinated biphenyl congeners. The PM was used for murine intratracheal instillation. DHA was purchased from TCI (Tokyo, Japan). Antibodies against cleaved Caspase-3 (catalog#9661), Bcl-2-associated X protein (Bax catalog#14796), anti-apoptotic protein B-cell lymphoma 2 (Bcl-2 catalog#2764), microtubule-associated protein 1 light chain 3-II (LC3-II catalog#43566), Beclin-1 (catalog#3495), rabbit anti-phospho-NF-κB p65 (Ser536; catalog#3033), and rabbit anti-NF-κB p65 (catalog#8242) were obtained from Cell Signaling Technology (MA, USA). The Enzyme-linked immunosorbent assay (ELISA) kits for defined antigens (IL-6, IL-8, IL-1β) were obtained from Boyun Biotechnology (Shanghai, China). The real-time polymerase quantitative chain reaction (RT-qPCR) primers were obtained from Sangon Biotech (Shanghai, China). All other RT-qPCR reagents were obtained from Takara Bio (Shiga, Japan).

### 2.2 Animal model of PM-induced lung injury

**Figure 1:**
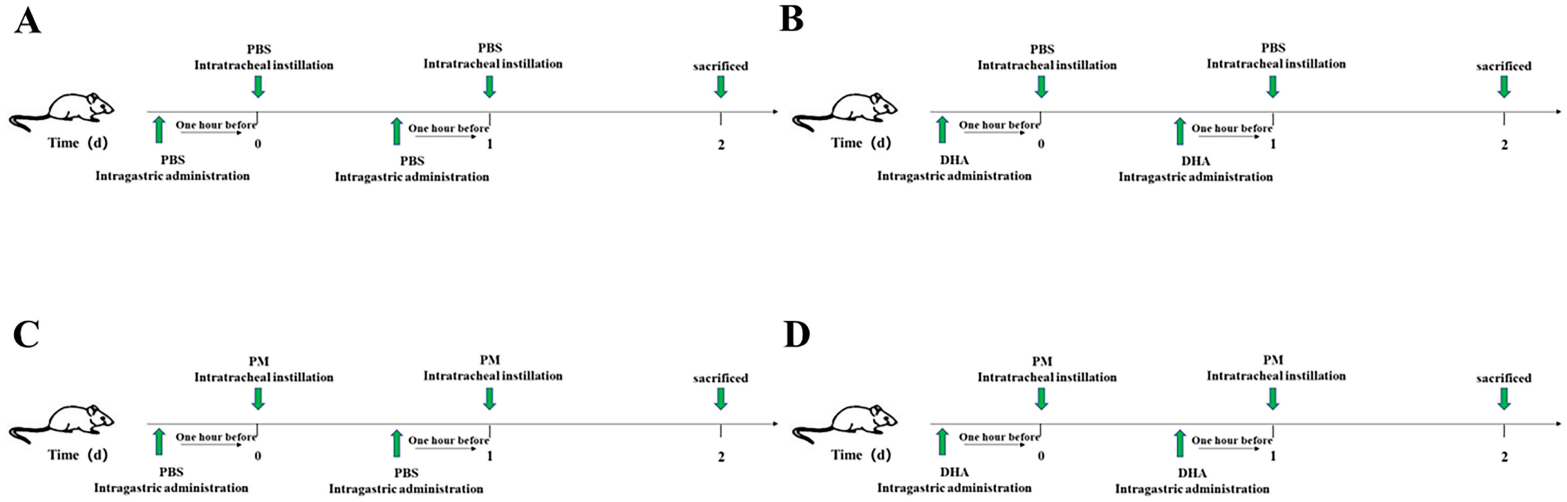
Schematic diagram of the *in vivo* experimental design. **(A)** Control group: Mice received intragastric administration of PBS followed by intratracheal instillation of PBS on two consecutive days. **(B)** DHA group: Mice received intragastric administration of DHA followed by intratracheal instillation of PBS on two consecutive days. **(C)** PM group: Mice received intragastric administration of PBS followed by intratracheal instillation of PM on two consecutive days. **(D)** PM+DHA group: Mice received intragastric administration of DHA followed by intratracheal instillation of PM on two consecutive days. All mice were sacrificed 24 h after the last instillation, and lung tissue and BALF were collected.

**Figure 2:**
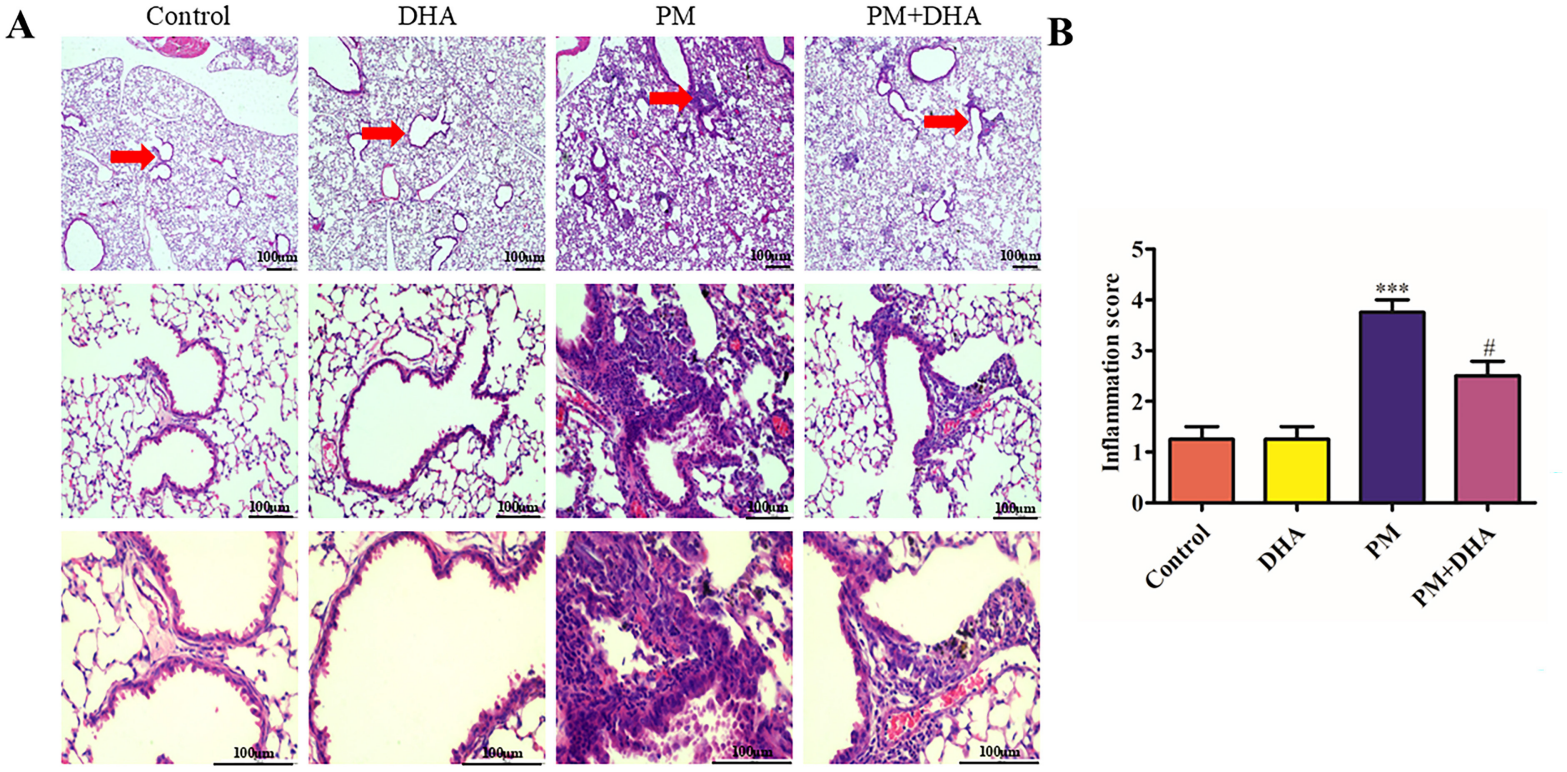
Pretreatment with DHA alleviates PM-induced lung injury *in vivo*. **(A)** The representative H&E stained lung sections. Scale bar = 100 μm. Arrows indicate inflammatory cell infiltration (red arrows) and structural damage in lung tissues. **(B)** The inflammatory scores for the H&E stained lung sections (*n* = 3/group). Data were presented as mean ± standard error of the mean (SEM, *n* = 3); *P < 0.05, **P < 0.01, ***P < 0.001 vs. Control group. ^#^*P* < 0.05, ^*##*^*P* < 0.01, ^*###*^*P* < 0.001 vs. PM group.

**Figure 3:**
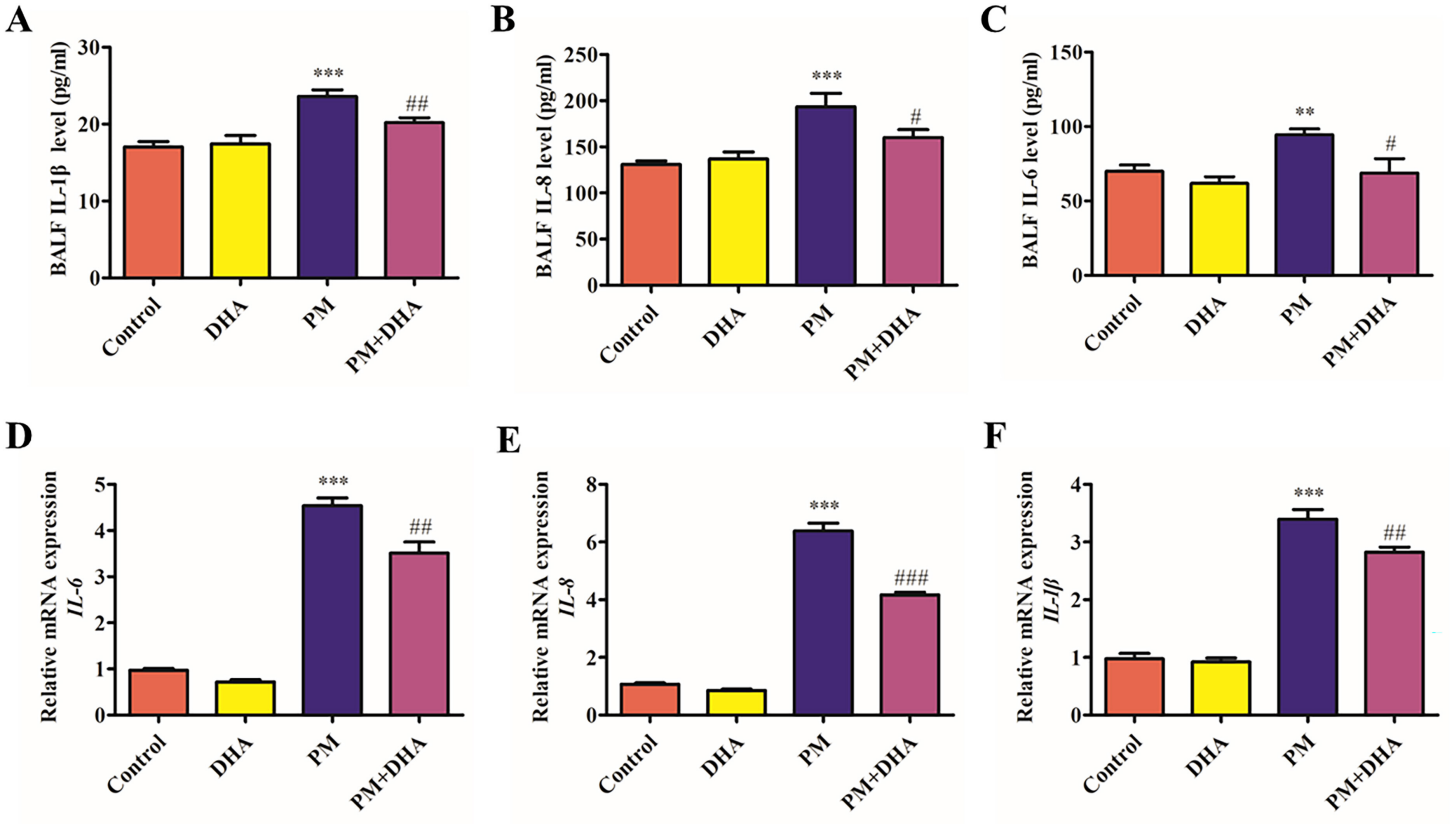
DHA pretreatment significantly reduces levels of IL-6, IL-8, and IL-1β in bronchoalveolar lavage fluid and cell supernatants, as quantified by ELISA and qRT-PCR. **(A–C)** The BALF IL-6, IL-8, and IL-1βprotein levels as quantified by ELISA. **(D–F)** The BALF IL-6, IL-8, and IL-1β relative mRNA expression levels as quantified by qPCR. Data were presented as mean ± standard error of the mean (SEM, *n* = 3); *P < 0.05, **P < 0.01, ***P < 0.001 vs. Control group. ^#^*P* < 0.05, ^*##*^*P* < 0.01, ^*###*^*P* < 0.001 vs. PM group.

**Figure 4:**
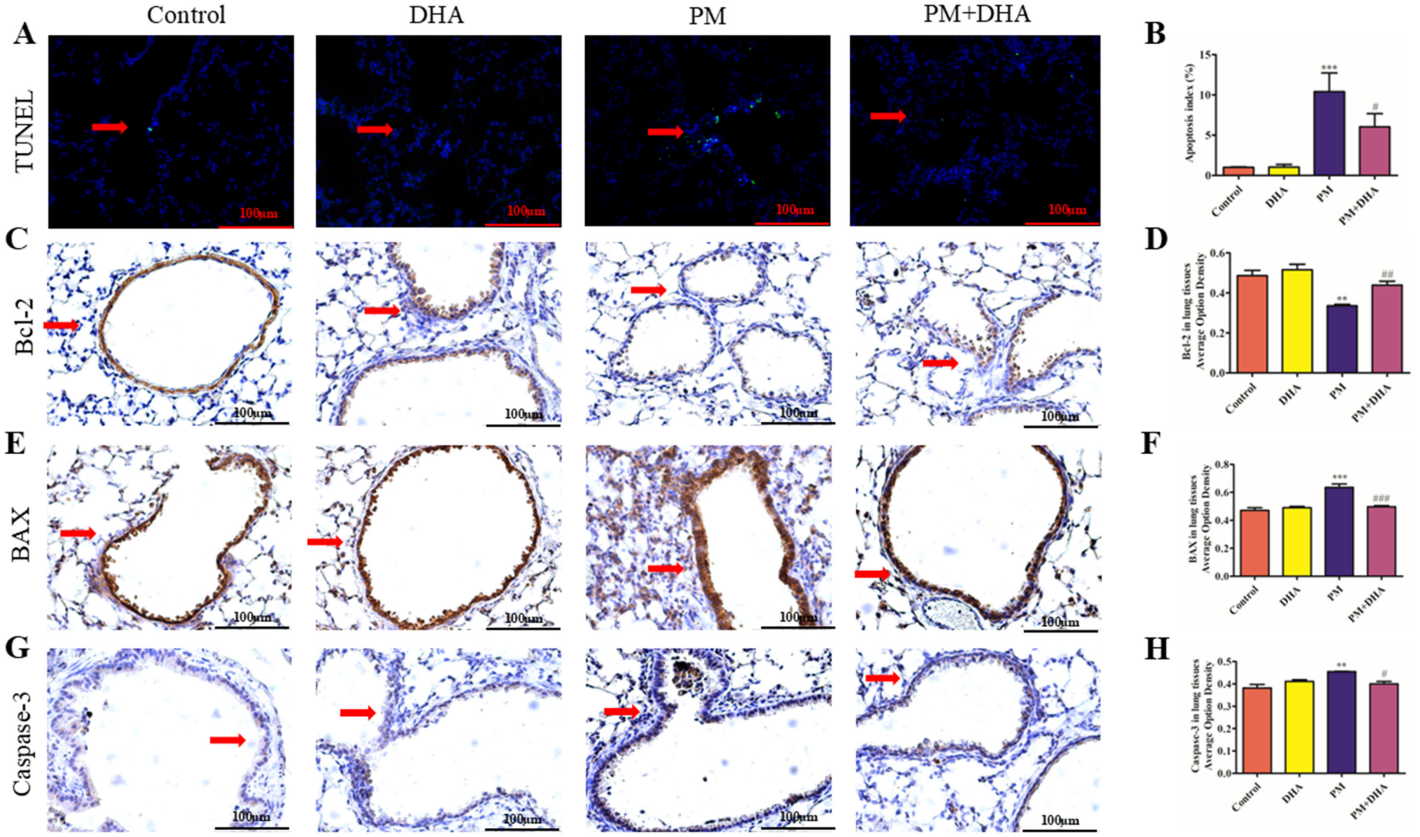
Pretreatment with DHA reduces apoptosis of lung tissue via regulation of pro-apoptotic proteins. Scale bar = 100 μm. **(A, B)** The representative sections of nuclear DNA fragmentation staining using TUNEL in the different groups, and the quantitative analysis of the number of TUNEL-positive airway epithelial cells. The IHC of BCL-2 **(C)**, Bax **(E)** and cleaved-Caspase-3 **(G)** of lung tissue sections obtained from the indicated groups, and the quantification of BCL-2 **(D)**, Bax **(F)** and cleave-caspase 3 **(H)** expression in lung tissue are shown. The results are presented as mean ± standard error of the mean (SEM; **P < 0.01 vs. Vehicle group; ^*##*^*P* < 0.01 vs. PM group, *n* = 3). *P < 0.05, **P < 0.01, ***P < 0.001 vs. Control group. ^#^*P* < 0.05, ^*##*^*P* < 0.01, ^*###*^*P* < 0.001 vs. PM group.

Male C57BL/6 mice (*n* = 40, 20–25 g) were purchased from the Beijing Vital River Laboratory Animal Technology Company (Beijing, China). These animals were housed in a standard pathogen-free environment, with unrestricted food and water. All protocols for animal experiments were approved by the Institutional Animal Care and Use Committee of the first affiliated hospital of Wenzhou Medical University (License number: 2022–0026). To ensure animal welfare, all procedures were conducted in accordance with the National Institutes of Health Guide for the Care and Use of Laboratory Animals. Animals were closely monitored daily for signs of distress or discomfort, and humane endpoints were applied to minimize suffering. At the end of the experiment, euthanasia was performed using sodium pentobarbital (150 mg/kg, intraperitoneally), ensuring a painless and humane process. The sample size of 10 mice per group was determined based on a power analysis using G^*^Power 3.1 software, assuming an alpha level of 0.05, a power of 0.8, and a medium effect size (Cohen's *f* = 0.5). This ensured sufficient statistical power to detect significant differences in key outcomes, such as Szapiel scores and cytokine levels. Then, these animals were randomized into four treatment groups: (1) Control group, mice received intragastric administration injection of PBS at 1 h before the intratracheal instillation of PBS at the same dose as the PM group (Figure 1A); (2) DHA group, mice re ceived intragastric administration injection of DHA (90 mg/kg/) at 1 h before the intratracheal instillation of PBS (Figure 1B); (3) PM group, mice received at intragastric administration injection of DHA 1 h before intratracheal instillation of PM (4 mg/kg; Figure 1C); (4) PM+DHA group, mice received intragastric administration injection of DHA at 1 h before treatment with PM (Figure 1D). The DHA and PM + DHA groups received intragastric administration injection of DHA at a dose of 90 mg/kg/d for 2 consecutive days, while the control mice received saline solution. At 1 h after these administrations, the PM (4 mg/kg) was intratracheally instilled to the model lung injury, while the control mice were instilled with PBS. After two consecutive days of PM instillation, these mice were sacrificed, and the lung tissue and the lung tissue and bronchoalveolar lavage fluid (BALF) were collected.

### 2.3 Cell culture

The Human bronchial epithelial cells (HBECs) were obtained from the Chinese Academy of Sciences (Shanghai, China), and grown in RPMI-1640 medium (Hyclone, UT, USA) containing 10% fetal bovine serum (Gibco, MA, USA) and penicillin/streptomycin (Gibco) at 37°C with 5% CO2. Based on preliminary experiments in our laboratory, 5 μM DHA was selected for its efficacy in reducing PM-induced inflammatory responses without cytotoxicity. Cells were pretreated with DHA (5 μM) for 1 h, followed by treatment with PM (200 μg/mL) or PBS for 24 h. Then, the RNA was collected for RT-qPCR.

### 2.4 RT-qpcr

TRIzol (Invitrogen, CA, USA) was used to isolate the cellular RNA, and the cDNA was prepared based on the provided protocols. The relative expression of murine IL-6, IL-8, and IL-1β was quantified via the ΔCt approach, and GAPDH was used for normalization. We analyzed these samples in triplicate using the primers, as follows: IL-6 (sense, 5′-TTCGGTCCAGTTGCCTTCT-3′; anti-sense, 5′-GGTGAGTGGCTGTCTGTGTG-3′), IL-8 (sense, 5′-TTGCCAAGGAGTGCTAAAGAA-3′; anti-sense, 5′-TTGCCAAGGAGTGCTAAAGAA-3′), IL-1β: (sense, 5′-ACTCCTTAGTCCTCGGCCA-3′, anti-sense, 5′-CCATCAGAGGCAAGGAGGAA-3′), and GAPDH (sense, 5′-AGGTCGGTGTGAACGGATTTG-3′; anti-sense, 5′-TGTAGACCATGTAGTTGAGGTCA -3′).

### 2.5 BALF collection

Mice were euthanized via 4% chloral hydrate, and eyeball extraction was conducted to mediate exsanguination. Then, the BALF was collected with a tracheal cannula, with 1 mL of PBS being gradually instilled into and withdrawn from the lung for three times. After collection, the BALF samples were centrifuged at 12,000 rpm for 15 min at 4°C.

### 2.6 ELISA

For ELISA, the BALF was carefully collected through a tracheal cannula, with 1 ml of PBS instilled into the lung. The collected BAL was then centrifuged at 12,000 rpm for 15 min at 4°C. The levels of IL-6, IL-8 and IL-1β in the BALF supernatants and cell culture supernatants were quantified using the indicated ELISA kits, according to the manufacturer's instructions.

### 2.7 Hematoxylin and eosin staining (H&E)

For histopathology, the lung tissues were fixed with 4% paraformaldehyde for more than 24 h, washed in PBS and dehydrated in increasing concentration of ethanol. Then, the lungs (*n* = 3 per group) were embedded in paraffin and prepared into 5 μm slices. Afterwards, these sections were de-paraffinized, rehydrated, H&E stained, and analyzed using a light microscope. The lung pathological injury score criteria were as follows: 1, no inflammation; 2, occasionally inflammatory cell infiltration; 3, most bronchi or blood vessels are covered with a thin layer. Inflammatory cell layer (1 to 5 layers); 4, sur of most bronchi or blood vessels There is a thick layer (> 5 layers) of inflammatory cells around. Three sections per specimen were stained and evaluated for Szapiel scoring, and three independent pathologists, who were blinded to the experiment, assessed the results.

### 2.8 Apoptosis assay

Apoptotic cells in lung tissue were determined using Terminal deoxynucleotidyl transferase dUTP nick-end labeling (TUNEL) staining with an *In Situ* Cell Death Detection Kit (Roche, Indianapolis, IN, USA), following the manufacturer's protocol. In brief, paraffin-embedded lung sections (6 μm) were dewaxed and rehydrated. After antigen retrieval in 0.1 M sodium citrate buffer (pH 6.0), the sections were incubated with TUNEL reaction mixture (Vial 1: Vial 2 = 1: 9) for 60 min at 37°C in a humidified atmosphere in the dark. To identify nuclei, tissues were counterstained with the fluorescent dye 4,6-diamidino-2-phenylindole (DAPI) for 5 min. All images were captured using a fluorescence microscope (Nikon, Tokyo, Japan). The number of TUNEL positive cells was counted under ×200 microscopic fields and expressed as cells per field. At least 10 fields of each mouse and six mice in each group were counted in a blinded manner.

### 2.9 Immunohistochemistry and immunofluorescent staining

The slides were incubated with antibodies against cleaved-Capase-3, Bax, Bcl-2 at 4°C overnight and stained with Diaminobenzidine (DAB) and counterstained with hematoxylin. The slides were then subjected to gradient ethanol dehydration, dimethyl benzene transparent, and mounted with Neutral resin cover slides. Images were captured using a Nikon ECLPSE 80i. For immunofluorescent staining, 5 μm sections were incubated at 4°C overnight with primary antibody against LC3-II, Beclin-1, p-p65, respectively. The slides were then incubated with donkey anti-rabbit secondary antibodies or donkey anti-mouse IgG-PE secondary antibodies for 1 h at room temperature. Then, DAPI was used for nuclear staining for 10 min, followed by fluorescent microscopic imaging.

### 2.10 Statistical analysis

All of the data were expressed as mean ± SEM (Standard Error of Mean). *In vitro* experiments were conducted with n ≥ 3 independent replicates, and the *in vivo* experiments were conducted with *n* = 10 mice per group. Statistical analyses were conducted using GraphPad Prism 9.0 software (GraphPad, San Diego, CA, USA). Prior to hypothesis testing, the normality of data distribution was assessed using the Shapiro-Wilk test, and homogeneity of variances was evaluated using Levene's test. For comparisons between two groups, the student's *t*-test (two-tailed) was used for normally distributed data with equal variances, while the Mann-Whitney U test was applied for non-normally distributed data. For comparisons among multiple groups, one-way ANOVA followed by Tukey's *post-hoc* test was used for normally distributed data with equal variances, and Welch's ANOVA was applied when variances were unequal. If assumptions of normality or homogeneity of variances were violated, non-parametric tests were applied as appropriate. A *P* < 0.05 was considered statistically significant. The choice of statistical tests was based on the type of data, experimental design, and distribution properties, ensuring appropriate and accurate analysis of the results.

## 3 Results

### 3.1 DHA alleviates PM-induced lung injury *in vivo*

We investigated the protective effects of DHA in a mouse model of PM-induced lung injury as depicted. Structural injury in lung samples from different treatment groups (*n* = 3 mice per group) was assessed using H&E staining, which revealed distinct morphological lesions and alterations in the lung tissue sections. There were no discernible differences between Control and DHA group. PM exposure was associated with a marked increase in acute lung inflammation, when compared to the lungs of Control group. This inflammation was characterized by a significant increase in inflammatory cell accumulation in the airways and alveoli. In contrast, mice pretreated with DHA exhibited a significant reduction in inflammatory cell infiltration into the lungs (Figure 2A).

Compared with that of the control group, the Szapiel scores of PM group increased significantly (*P*<*0.001 95%CI* −*3.593 to* −*1.407*). Compared with that of the PM group, the Szapiel score of the PM+DHA group decreased significantly (*P* = *0.02 95%CI 0.1575 to 2.343*, Figure 2B). These findings demonstrate that DHA therapy exerts protective effect.

### 3.2 DHA inhibits production of proinflammatory cytokines in PM-induced lung injury *in vivo* and *in vitro*

To reveal the role of DHA in the PM-induced lung injury model, we investigated whether DHA inhibits production of proinflammatory cytokines in the BALF and HBECs. we used an ELISA and qRT-PCR to detect inflammatory cytokines. The protein levels of IL-6, IL-8, IL-1β in the BALF increased after PM stimulation (*P* = *0.04 95%CI* −*47.47 to* −*1.315; P*<*0.001 95%CI* −*98.77 to* −*26.34; P*<*0.001 95% CI* −*9.776 to* −*3.375*) but decreased after DHA treatment significantly (*P* = *0.03 95%CI 2.486 to 48.65; P* = *0.08 95%CI* −*2.770 to 69.65; P* = *0.03 95%CI 0.2197 to 6.621*, Figures 3A–C). Consistent with the ELISA results, the qRT-PCR result demonstrated that stimulation with PM significantly increased the mRNA levels of interleukin IL-6, IL-8, IL-1β (*P*<*0.001 95% CI* −*4.250 to* −*2.892;P*<*0.001 95%CI 0.3516 to 1.710;P*<*0.001 95%CI* −*2.921 to* −*1.920*, Figures 3D–F), while DHA pretreatment was sufficient to significantly reduce these levels upon PM stimulation (*P* = *0.005 95%CI 0.3516 to 1.710;P*<*0.001 95%CI 0.3516 to 1.710; P* = *0.03 95%CI 0.07184 to 1.073*). These data suggested that DHA exerts an anti-inflammatory effect in lung tissue and cells, and this effect may account for its therapeutic role in PM-induced lung injury.

### 3.3 DHA reduces apoptosis of lung tissue via regulation of pro-apoptotic proteins

TUNEL staining was performed to assess the levels of apoptosis in lung tissue. As shown in Figures 4A, B, compared to the Control group, the number of TUNEL-positive cells in PM group was dramatically increased. Significantly (*P*<*0.001 95%CI* −*15.06 to* −*3.751*), the proportion of TUNEL-positive cells was much lower in PM+DHA group (*P* = *0.17 95%CI* −*1.284 to 10.02*). The results indicated that DHA treatment protected lung tissue from PM-induced apoptosis based on TUNEL staining. In order to explore the mechanisms underlying these effects, we examined the expression of pro-apoptotic proteins involved in regulation of cell apoptosis (BCL-2, Bax) and cleaved-Caspase-3 by IHC staining (Figures 4C–H). The expression of Bax and cleaved-Caspase-3 were significantly increased upon PM injury (*P*<*0.001 95%CI* −*0.2418 to 0.08820; P* = *0.004 95%CI* −*0.1177 to* −*0.02698*), whereas BCL2 expression was decreased. Significantly (*P* = *0.005 95%CI 0.05235 to 0.2483*). DHA treatment inhibited the pro-apoptotic expression/activation of Bax/BCL2 (*P* = *0.002 95%CI 0.06087 to 0.2145; P* = *0.04 95%CI* −*0.2016 to* −*0.005685*) and cleaved-Caspase-3 (*P* = *0.02 95%CI 0.008982 to 0.09968*), respectively. The results suggest that DHA protects lung injury from PM-induced apoptosis via regulation of pro-apoptotic proteins.

### 3.4 DHA downregulates the autophagy level in PM-induced lung injury model

**Figure 5:**
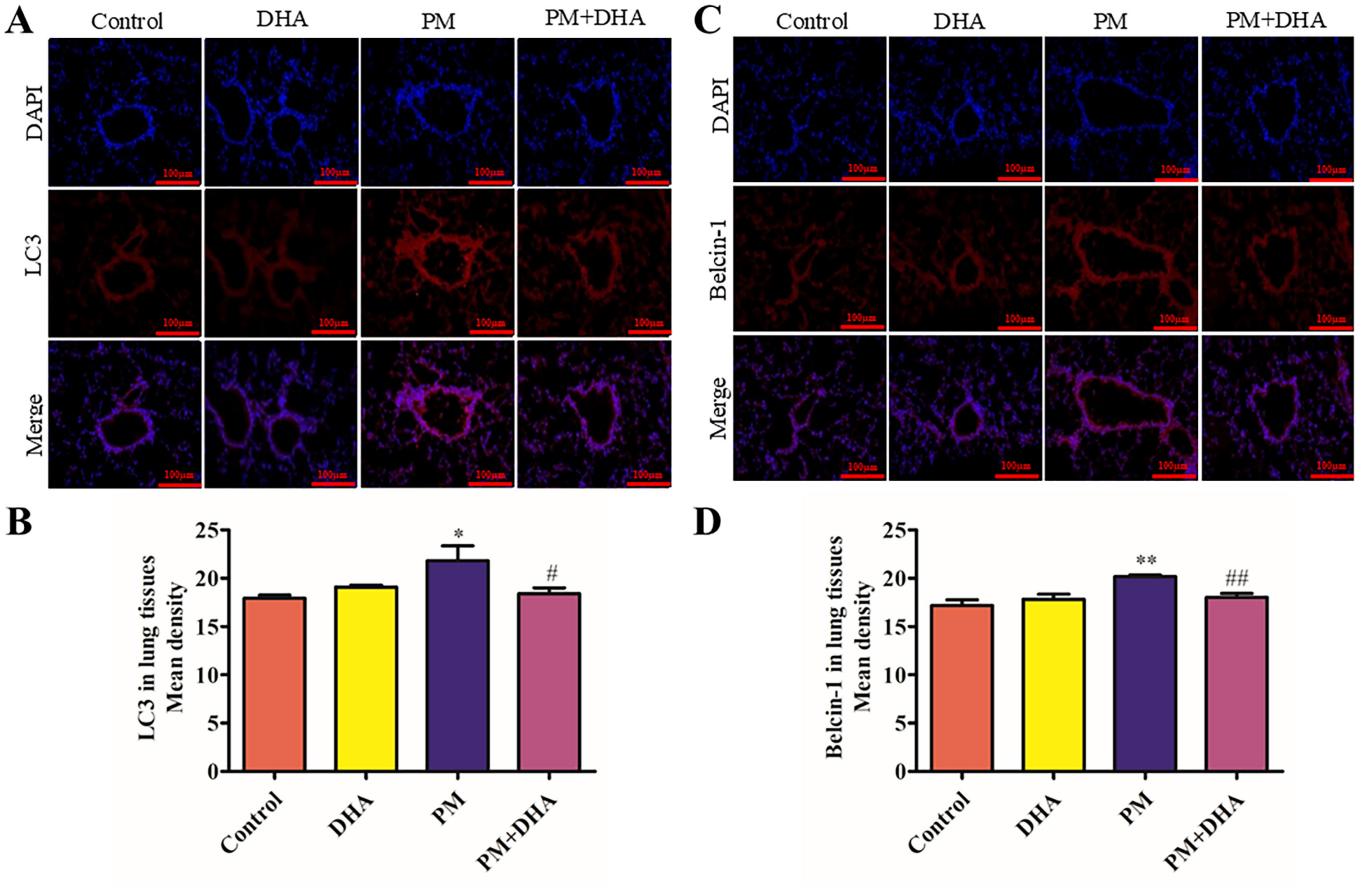
Pretreatment with DHA downregulates the autophagy level in PM-induced lung injury model. At 2 days post-PM exposure, the immunofluorescent detection of lung LC3 (**A**, **B**, scale bars = 100 μm) and Beclin-1 (**C**, **D**) was conducted. DAPI (blue) was used for nuclear staining (scale bars = 50 μm). These images were taken using a BX51 microscope DP71 camera at ×200 magnification. *P < 0.05, **P < 0.01, ***P < 0.001 vs. Control group. ^#^*P* < 0.05, ^*##*^*P* < 0.01, ^*###*^*P* < 0.001 vs. PM group.

Autophagy is known to play a crucial role in the etiology of lung injury caused by PM exposed. This prompted us to further study whether autophagy participates in the protection function of DHA. Detection of LC3I to LC3II conversion and expression of Beclin-1 remains the most reliable methods to gauge autophagic activity. Co-detection of Beclin-1 and LC3 by immunofluorescence staining revealed that the LC3-II and Beclin-1 levels dramatically increased in the PM group compared to Control group (*P* = *0.05 95%CI* −*7.759 to* −*0.07269; P* = *0.007 95%CI* −*5.058 to* −*0.9575)*, but greatly reduced by DHA (*P* = *0.08 95%CI* −*0.4110 to 7.275; P* = *0.04 95% 0.1332 to 4.233*, Figures 5A–D). These results indicate that DHA may improve PM-induced lung injury by regulating the autophagy level.

### 3.5 DHA suppresses the activation of NF-κb signaling in the lung tissue of mice treated with PM

**Figure 6:**
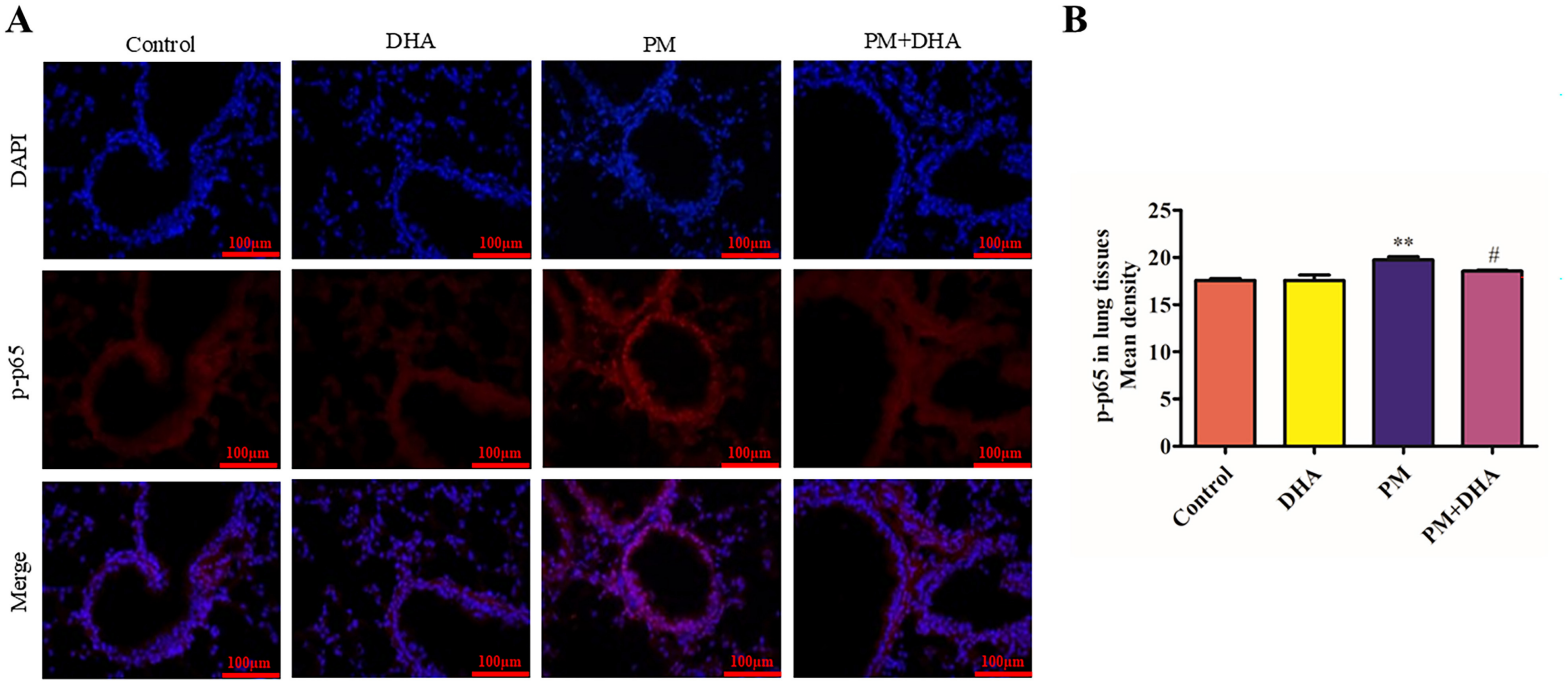
Pretreatment with DHA suppresses the activation of NF-κB signaling in the lung tissue of mice treated with PM. The immunofluorescent detection of lung p-p65 (scale bars = 100 μm) was conducted. **(A)** DAPI (blue) was used for nuclear staining (scale bars = 50 μm). **(B)** Quantification of p-p65 in lung tissue fluorescence staining. These images were taken using a BX51 microscope DP71 camera at ×200 magnification. *P < 0.05, **P < 0.01, ***P < 0.001 vs. Control group. ^#^*P* < 0.05, ^*##*^*P* < 0.01, ^*###*^*P* < 0.001 vs. PM group.

NF-κB signaling pathway is closely related to the abnormal release of pro-inflammatory cytokines (16). To explore the mechanism of DHA in protecting against PM-induced lung injury, we further investigated the effect of DHA on the activation of the NF-κB signaling pathway in lung tissue. immunofluorescence staining exhibited the number of cells with nuclear location of p-p65 were extremely increased after PM exposed (*P* = *0.010 95%CI* −*3.746 to* −*0.5909*), suggesting the activation of NF-κB signaling pathway. Notably, DHA treatment significantly blocked p-p65 nuclear location induced by PM (*P* = *0.15 95%CI* −*0.3917 to 2.764*, Figures 6A, B). These data indicated that DHA ameliorated PM-induced inflammation possibly by blocking NF-κB signaling activation.

## 4 Discussion

PM-related diseases, including chronic obstructive pulmonary disease (COPD), asthma, and lung cancer, impose a substantial global health and economic burden. PM2.5 exposure is responsible for millions of premature deaths annually and significant societal costs, such as increased healthcare expenditures and reduced workforce productivity, particularly in regions with severe air pollution (18). These challenges highlight the urgent need for effective therapeutic strategies. In this context, the findings of this study explores how DHA protects against PM-induced lung injury in a murine model, highlighting its potential as a therapeutic agent. DHA demonstrated a significant reduction in lung inflammation, as evidenced by decreased inflammatory cell infiltration and lower Szapiel scores in DHA-pretreated groups compared to those exposed solely to PM. These results are consistent with prior studies indicating DHA's anti-inflammatory properties in various disease models. For instance, DHA has been shown to mitigate inflammation in models of acute kidney injury and chronic airway inflammation by inhibiting the NF-κB pathway and reducing pro-inflammatory cytokines (9). Our study extends these findings by demonstrating DHA's capacity to attenuate lung inflammation induced by environmental pollutants, such as PM, thereby preventing structural damage and preserving lung function. This protective effect is particularly relevant given the global burden of respiratory diseases exacerbated by air pollution and underscores the potential of DHA as a multifaceted therapeutic compound in the context of respiratory pathologies linked to environmental exposures.

**Figure 7:**
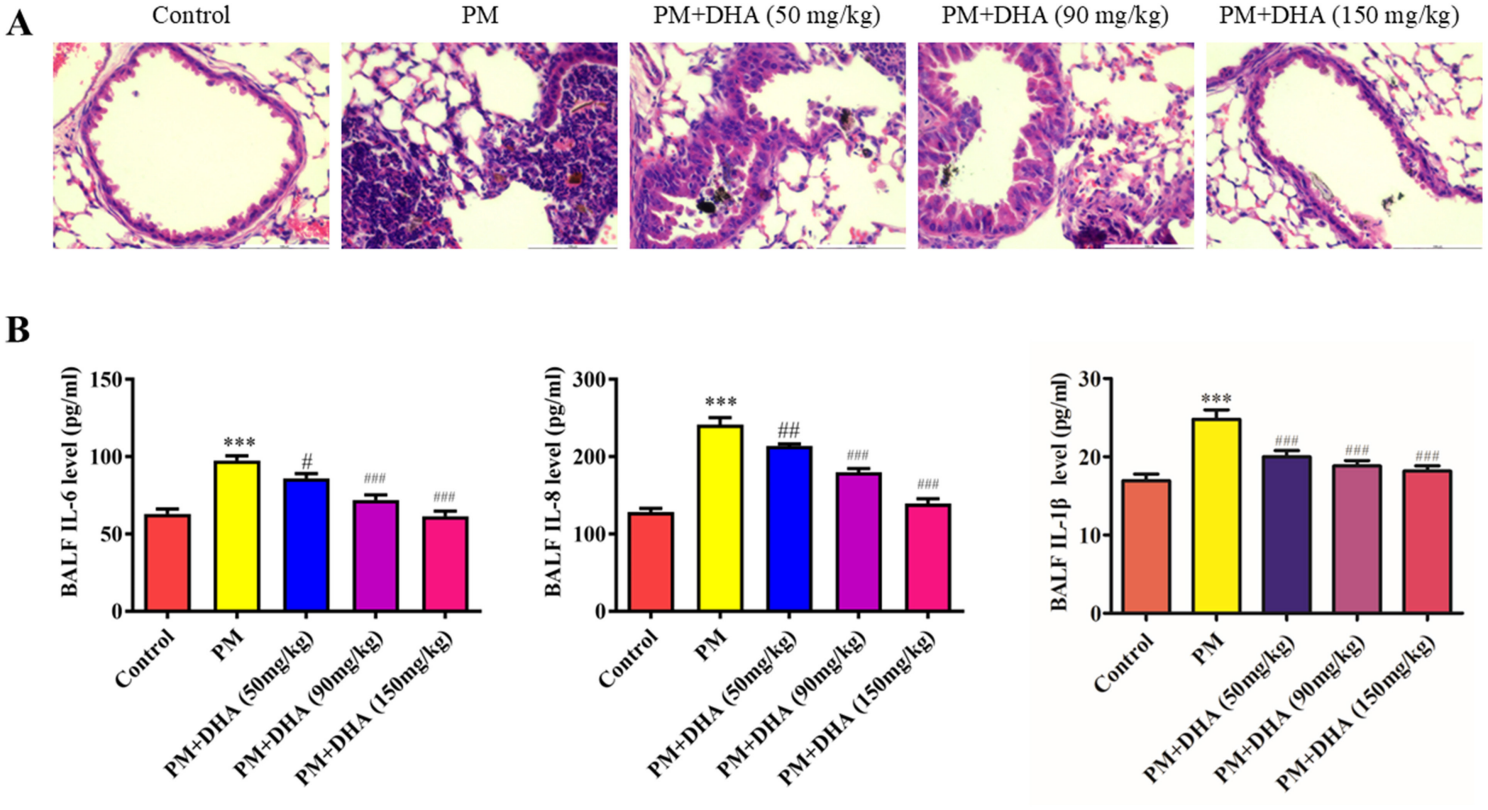
Dose-dependent effects of DHA on PM-induced lung injury. **(A)** Representative histological sections of lung tissues with varying DHA doses. **(B)** Quantitative analysis of inflammatory cytokine levels in different dose groups. *P < 0.05, **P < 0.01, ***P < 0.001 vs. Control group. ^#^*P* < 0.05, ^*##*^*P* < 0.01, ^*###*^*P* < 0.001 vs. PM group.

Furthermore, our investigation into the anti-inflammatory effects of DHA in both *in vivo* and *in vitro* settings revealed a significant downregulation of pro-inflammatory cytokines, including IL-6, IL-8, and IL-1β, following DHA treatment. This outcome aligns with existing literature, which documents the inhibitory effects of DHA on the NF-κB signaling pathway, a key regulator of inflammation and immune responses (19, 20). By attenuating the production of these cytokines, DHA addresses the fundamental inflammatory processes that exacerbate PM-induced lung injury. To clarify the dose-dependent effects of DHA, we compared different doses in PM-induced lung injury (Figure 7). DHA treatment progressively reduced inflammation and improved lung structure, with higher doses showing greater protective effects. These findings suggest a dose-dependent relationship, supporting its potential for clinical application. Notably, TNF-α, a major upstream mediator of inflammatory signaling, has also been implicated in PM-induced injury. Although not directly measured in this study, it is plausible that DHA's suppression of NF-κB activation also reduces TNF-α levels, as reported in other models of inflammation (21). Furthermore, PM exposure is known to elevate reactive oxygen species (ROS), which exacerbate oxidative stress and amplify inflammatory responses. DHA has demonstrated antioxidant properties in prior studies by enhancing the activity of superoxide dismutase (SOD) and reducing ROS generation (22). These interactions suggest that DHA not only mitigates the cytokine-mediated inflammatory cascade but also modulates broader oxidative and inflammatory pathways. Expanding future analyses to include TNF-α and ROS could provide a more holistic view of the biological processes involved in DHA's protective effects against PM-induced lung injury.

In addition to its anti-inflammatory properties, DHA's role in modulating apoptotic pathways presents a critical mechanism for its protective effects against PM-induced lung injury. Our study demonstrated that DHA treatment significantly reduced the expression of pro-apoptotic proteins such as Bax and cleaved-Caspase-3, while upregulating the expression of the anti-apoptotic protein Bcl-2. These findings are indicative of DHA's ability to inhibit apoptosis in lung tissues, a vital aspect of its protective action. Previous research has established the involvement of apoptosis in the pathogenesis of lung injury, where excessive apoptotic cell death contributes to tissue damage and inflammation (23, 24). This modulation of the Bax/Bcl-2 ratio likely stabilizes the mitochondrial membrane potential and inhibits the release of cytochrome c, which is a key step in the intrinsic apoptotic pathway. Consequently, the activation of Caspase-3, a critical effector in the apoptotic cascade, is reduced, mitigating cell death in lung tissues. These findings are consistent with prior studies that identified DHA as a regulator of apoptosis in cancer and neurodegenerative models, underscoring its versatility as a therapeutic agent (25). By counteracting this process, DHA not only prevents cell loss but also curtails the subsequent inflammatory response, underscoring a dual therapeutic strategy that targets both cell death and inflammation. While apoptosis has been extensively studied in the context of other diseases, this study contributes novel insights into how DHA modulates apoptotic processes in response to PM exposure, expanding its relevance to environmental health. This regulation of apoptotic pathways by DHA could be a pivotal factor in its efficacy against lung damage induced by environmental pollutants and warrants further investigation into its potential as a preventive and therapeutic agent in respiratory diseases characterized by apoptosis and inflammation.

The modulation of autophagy by DHA also emerged as a significant aspect of its protective mechanism against PM-induced lung injury. Our results showed that DHA effectively downregulated the levels of LC3-II and Beclin-1, markers of autophagic activity, in lung tissues exposed to PM. This regulation may play a critical role in mitigating lung injury, as autophagy is known to have a dual function in cellular homeostasis and stress response, providing both protective and pathological effects depending on the context (26). These findings are consistent with prior studies showing that DHA modulates autophagy in diverse disease contexts. For instance, in cancer models, DHA has been reported to induce autophagy through ROS generation and AMPK activation, facilitating cell death in tumor cells (22). Similarly, in neurodegenerative diseases, DHA enhances autophagy via the mTOR signaling pathway, promoting the clearance of damaged organelles and reducing neuronal toxicity (27). However, our study highlights a different aspect of DHA's action, as it suppresses excessive autophagic activity in the context of PM-induced lung injury, thereby restoring autophagic balance and preventing tissue damage. This suggests that DHA's effects on autophagy may be context-dependent, modulating autophagy levels to either promote cell survival or induce cell death depending on the pathological condition. It is plausible that DHA influences upstream signaling pathways, such as mTOR or AMPK, to restore autophagic balance under conditions of PM-induced stress. These pathways are known to play crucial roles in autophagic regulation, and future studies should aim to clarify the precise molecular interactions involved. While autophagy is generally considered a protective mechanism against cellular stress, dysregulated autophagy has been implicated in the pathogenesis of various diseases, including lung injury, where it can contribute to cell death and inflammation. The reduction in autophagic markers following DHA treatment indicates a restoration of autophagic balance, potentially preventing the deleterious effects of excessive autophagy in the context of PM exposure. This finding adds a new dimension to the understanding of DHA's multifaceted protective effects, suggesting that its therapeutic potential extends beyond anti-inflammatory and anti-apoptotic actions to include the regulation of autophagic processes. Further studies are warranted to elucidate the precise mechanisms by which DHA modulates autophagy in the setting of lung injury and to explore its implications for therapeutic interventions in diseases characterized by autophagic dysregulation.

Additionally, our investigation highlighted DHA's capacity to suppress the activation of the NF-κB signaling pathway in lung tissues subjected to PM exposure. This pathway plays a central role in mediating inflammatory responses and has been identified as a target for therapeutic intervention in inflammatory diseases. DHA's inhibition of the NF-κB pathway may involve suppression of IκB kinase (IKK) activation, which prevents the degradation of IκBα and subsequent nuclear translocation of p65, as suggested by previous studies (28). This reduction in NF-κB activity likely downregulates the transcription of pro-inflammatory cytokines such as IL-6, IL-8, and IL-1β, mitigating the inflammatory cascade triggered by PM exposure. The suppression of NF-κB signaling is particularly significant, as this pathway regulates the expression of numerous genes involved in inflammation, immune responses, and cell survival. By inhibiting this pathway, DHA not only reduces the production of pro-inflammatory cytokines but also influences other downstream processes that contribute to lung injury and repair. The current study builds upon existing knowledge by directly linking DHA's modulation of NF-κB to improvements in both structural and molecular indicators of lung health in a pollution-induced injury model. These findings are consistent with existing research that emphasizes the importance of NF-κB in lung diseases and positions DHA as a promising candidate for modulating inflammatory responses in the lung. Given the complexity of NF-κB signaling and its pivotal role in various pathological conditions, further investigation into how DHA precisely modulates this pathway could unveil new therapeutic strategies for managing respiratory diseases associated with air pollution and inflammation.

In light of these comprehensive findings, the therapeutic potential of DHA against PM-induced lung injury underscores a promising avenue for future research and clinical applications. DHA's multifaceted protective mechanisms, encompassing anti-inflammatory, anti-apoptotic, autophagy-regulating, and NF-κB signaling suppression activities, suggest its utility as a comprehensive therapeutic agent in combating lung injuries induced by particulate matter. The global burden of diseases associated with air pollution, particularly in urban areas with high levels of PM, necessitates the exploration of effective treatment strategies. DHA, with its broad spectrum of biological activities, offers a potential therapeutic strategy that could be further optimized and tailored for specific respiratory conditions.

However, this study has limitations. First, mechanistic studies using alveolar epithelial cells (e.g., A549) or primary lung cells were not conducted. These models, which are directly exposed to PM in the lung, could provide additional insights into the cellular effects of DHA and its modulation of the NF-κB pathway. Second, the pharmacokinetics and potential off-target effects of DHA were not explored, and addressing these aspects in future studies would provide a more comprehensive understanding of its safety and efficacy. Future research should also focus on delineating the precise molecular pathways through which DHA exerts its protective effects, exploring its efficacy in clinical settings, and assessing its therapeutic potential in a wider range of pulmonary diseases. Beyond its potential as a standalone therapeutic agent, DHA could be integrated into existing treatment protocols for PM-related diseases. Its anti-inflammatory and anti-apoptotic properties may complement the effects of current anti-inflammatory drugs, while its regulation of autophagy could enhance antioxidant therapies. Moreover, DHA's low toxicity and oral bioavailability make it a promising candidate for long-term use in combination therapies. Clinical studies are needed to assess the synergistic effects of DHA with standard treatments, such as bronchodilators or corticosteroids, to improve patient outcomes. This approach could facilitate the inclusion of DHA into broader therapeutic strategies aimed at mitigating the respiratory impacts of air pollution.

## Data Availability

The raw data supporting the conclusions of this article will be made available by the authors, without undue reservation.
