# Supplementary material for: Dihydroartemisinin attenuates PM-induced lung injury by inhibiting inflammation and regulating autophagy
Source: Front Public Health. 2025 Mar 7;13:1548224. doi: 10.3389/fpubh.2025.1548224 (PMC11925939; doi:10.3389/fpubh.2025.1548224)
Supplement: Supplementary file 1 [file Data_Sheet_1.pdf]

Supplementary material

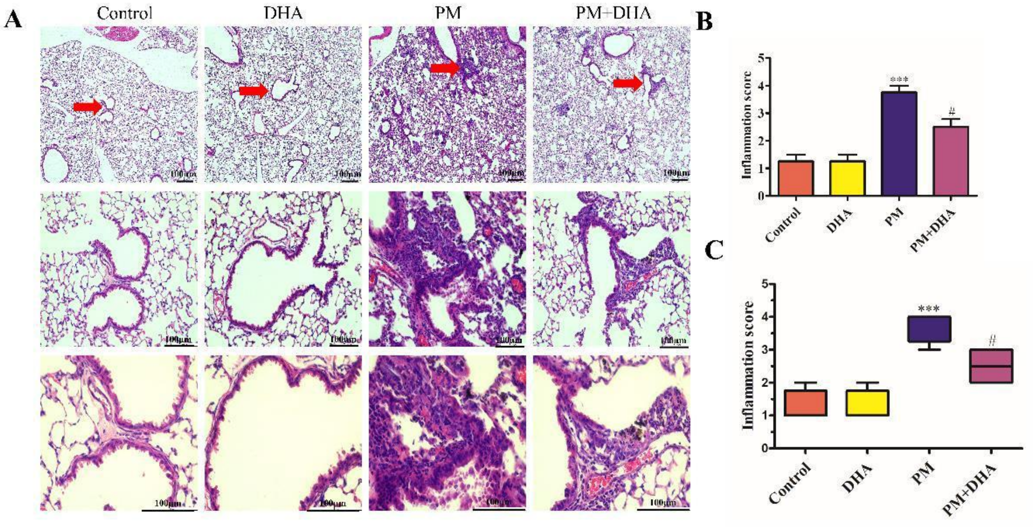

Figure 2. Pretreatment with DHA alleviates PM-induced lung injury in vivo. (A) The representative H&E stained lung sections. Scale bar = 100 μm. Arrows indicate inflammatory cell infiltration (red arrows) and structural damage in lung tissues. (B) The inflammatory scores for the H&E stained lung sections (n = 3/group). (C): The box plots of the inflammatory scores for the H&E stained lung sections (n = 3/group). Data were presented as mean ± standard error of the mean (SEM, n = 3); *P < 0.05, **P < 0.01 vs. control. #P < 0.05, ##P < 0.01vs. PM.

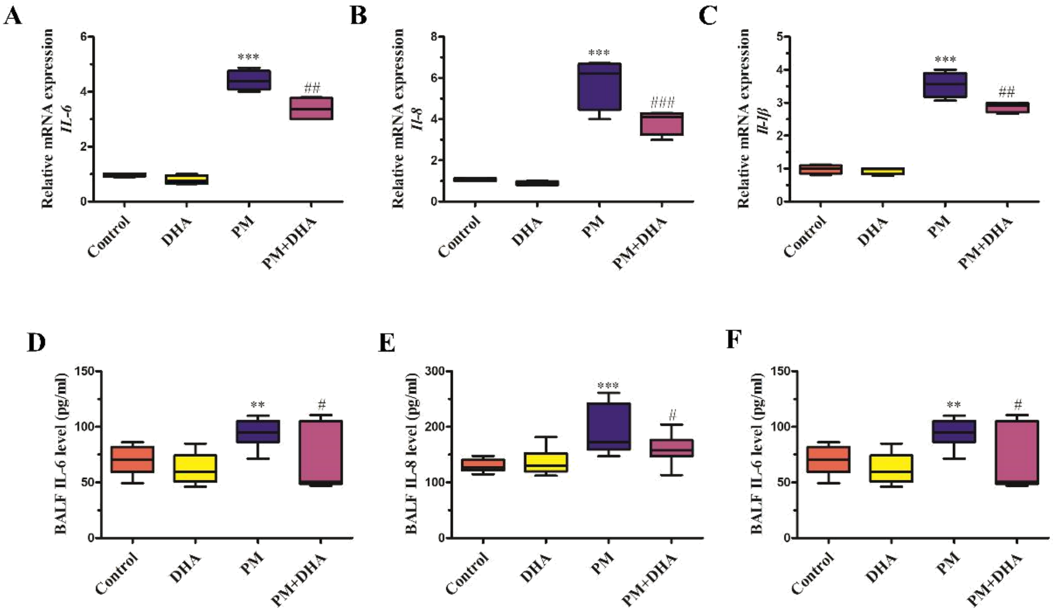

Figure 3. DHA pretreatment significantly reduces levels of IL-6, IL-8, and IL-1β in bronchoalveolar lavage fluid and cell supernatants, as quantified by ELISA and qRT-PCR. (A-C) The box plot of the BALF IL-6, IL-8 and IL-1βprotein levels as quantified by ELISA. (D-F) The box plot of the BALF IL-6, IL-8 and IL-1β relative mRNA expression levels as quantified by qPCR. Data were presented as mean ± standard error of the mean (SEM, n = 3); *P < 0.05, **P < 0.01 vs. control. #P < 0.05, ##P < 0.01vs. PM.

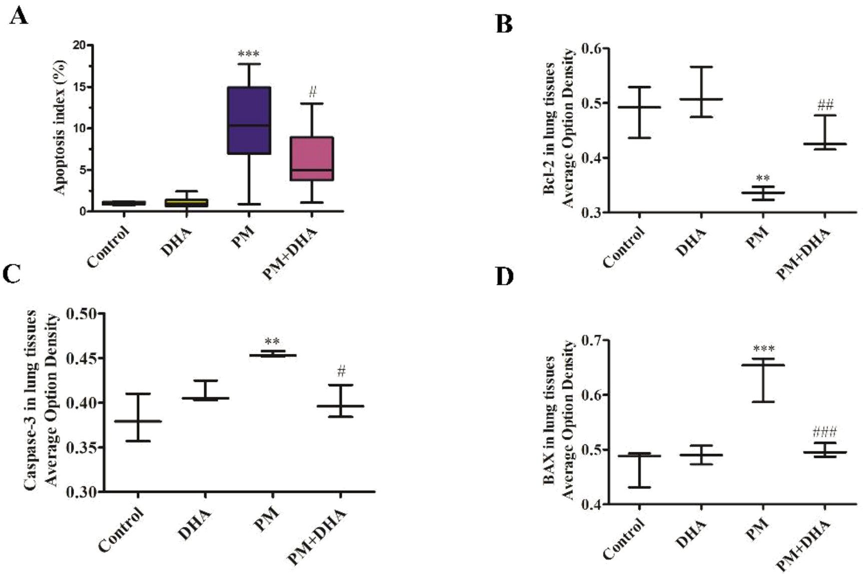

Figure 4 Pretreatment with DHA reduces apoptosis of lung tissue via regulation of pro-apoptotic proteins. The box plot of the quantitative analysis of the number of TUNEL-positive airway epithelial cells (A), and the quantification of BCL-2 (B), cleave-caspase 3(C) and Bax (D) expression in lung tissue are shown. The results are presented as mean ± standard error of the mean (SEM; **P < 0.01 vs Vehicle group; ##P < 0.01 vs PM group, n = 3).

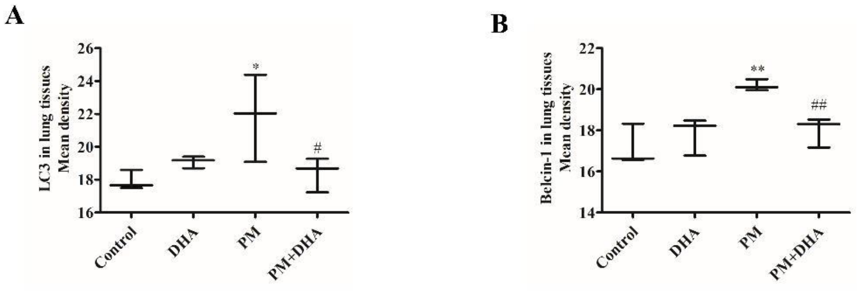

Figure 5. Pretreatment with DHA downregulates the autophagy level in PM-induced lung injury model. At two days post-PM exposure, the immunofluorescent detection of lung LC3 scale bars = 100 μm) and Beclin-1 was conducted..DAPI (blue) was used for nuclear staining (scale bars = 50 μm). The box plot of the quantitative analysis of LC3 (A) and Bclcin-1(B) in lung tissue fluorescence staining.

A

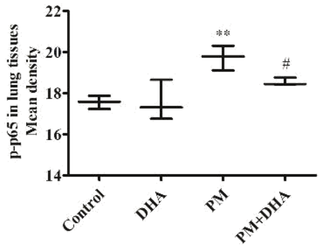

Figure 6. Pretreatment with DHA suppresses the activation of NF-κB signaling in the lung tissue of mice treated with PM. (A) The box plot of the quantification of p-p65 in lung tissue fluorescence staining.

A

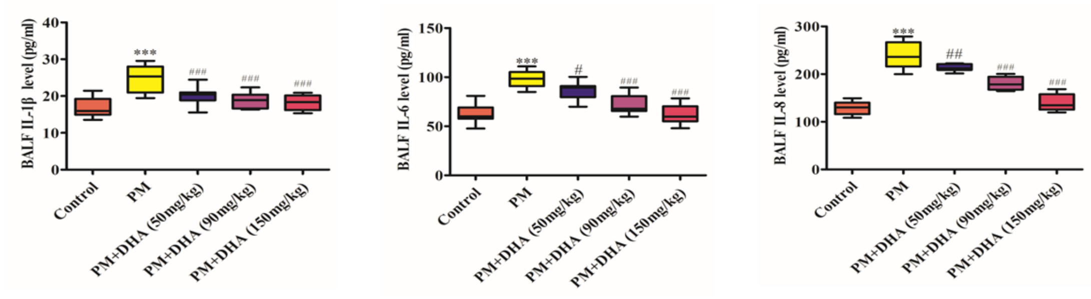

Figure 7. Dose-dependent effects of DHA on PM-induced lung injury.
(A) Quantitative analysis of inflammatory cytokine levels in different dose groups."
